# Automated Manipulation of Miniature Objects Underwater Using Air Capillary Bridges: Pick-and-Place, Surface Cleaning, and Underwater Origami

**DOI:** 10.1021/acsami.1c23845

**Published:** 2022-01-26

**Authors:** Tal Weinstein, Hagit Gilon, Or Filc, Camilla Sammartino, Bat-El Pinchasik

**Affiliations:** Faculty of Engineering, School of Mechanical Engineering, Tel-Aviv University, Tel-Aviv 6997801, Israel

**Keywords:** bubbles, 3D printing, robotic arm, underwater reversible adhesion, capillary bridges

## Abstract

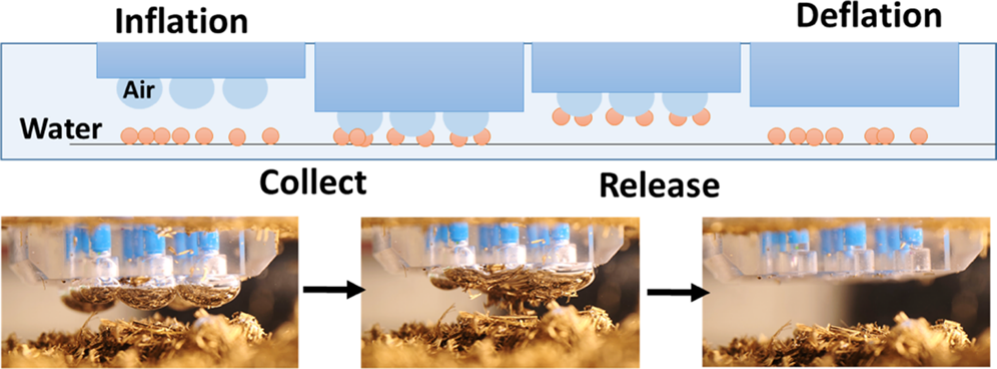

Various insects can entrap and stabilize air plastrons and bubbles underwater. When these bubbles interact with surfaces underwater, they create air capillary bridges that de-wet surfaces and even allow underwater reversible adhesion. In this study, a robotic arm with interchangeable three-dimensional (3D)-printed bubble-stabilizing units is used to create air capillary bridges underwater for manipulation of small objects. Particles of various sizes and shapes, thin sheets and substrates of diverse surface tensions, from hydrophilic to superhydrophobic, can be lifted, transported, placed, and oriented using one- or two-dimensional arrays of bubbles. Underwater adhesion, derived from the air capillary bridges, is quantified depending on the number, arrangement, and size of bubbles and the contact angle of the counter surface. This includes a variety of commercially available materials and chemically modified surfaces. Overall, it is possible to manipulate millimeter- to sub-millimeter-scale objects underwater. This includes cleaning submerged surfaces from colloids and arbitrary contaminations, folding thin sheets to create three-dimensional structures, and precisely placing and aligning objects of various geometries. The robotic underwater manipulator can be used for automation and control in cell culture experiments, lab-on-chip devices, and manipulation of objects underwater. It offers the ability to control the transport and release of small objects without the need for chemical adhesives, suction-based adhesion, anchoring devices, or grabbers.

## Introduction

Air bubbles and gas plastrons serve various aquatic and semiaquatic insects for respiration.^1^ In some cases, it has been suggested to enhance underwater reversible adhesion through controlled dewetting of submerged surfaces.^2^ These bubbles or plastrons are usually formed when the insect enters the water^3^ and is stabilized by small hair, very often hydrophobic, covering their body.^4^ Good adhesion of bubbles to insects’ bodies allows them not only to breathe underwater but also to perform locomotion and stabilize themselves at specific interfaces. For example, it allows backswimmers (*Notonectidae, Anisops*) to be trapped upside down at the water–air interface^5^ or fire ants (*Solenopsis invicta*) to join efforts and create floatable rafts by collecting bubbles underwater.^6^ These examples from nature demonstrate that bubbles comprise a powerful tool for reversible adhesion and placement of small or light objects at solid–water and water–air interfaces. Moreover, bubble adhesion is strong enough to overcome hydrodynamic forces underwater, resulting from insects’ locomotion.^7^ Such interfacial phenomena in nature can inspire robotic systems, small enough to be dominated by interfacial forces or to make use of them.^8−13^

Liquid capillary bridges in air regained considerable attention in the last decades since many insects rely on liquid secretion from their setae to attach to a variety of surfaces.^14−17^ Theoretically, it was demonstrated that scaling laws of multiple liquid bridges do not necessarily follow the liquid capillary adhesion equation,^18^ when a large number of bridges are involved.^19^ The mechanism of reversible adhesion using liquid capillary bridges was also used in droplet manipulation,^20,21^ small-scale devices,^22^ fabrication of particles,^23^ and patterning surfaces.^24^ However, the possibility to use air capillary bridges for automated manipulation of small objects underwater has not yet been fully investigated.

While hook- or claw-based cranes are efficient in picking bulky objects, they are not suitable for lifting multiple small objects or objects with a high aspect ratio. Vacuum-driven grippers, especially underwater, rely on the ability to seal the contact properly.^25−27^ This could not be achieved for surfaces with high roughness, complex geometry, very small features, or surfaces with holes.^28^ Synthetic biology has also proven itself for adhesion underwater, using mussel foot proteins.^29^ This adhesion mechanism, however, relies on chemicals, as opposed to physical reversible adhesion. Generally, there is a delicate interplay between the adhesion strength and reversibility, namely, ease of detachment.

Some geometries are especially difficult to grip and lift, such as thin sheets and long but slender rods. Very often, adhesion is discussed in terms of attachment strength but not for the use of transporting objects underwater. This is particularly relevant when multiple miniature objects need to be picked, placed, or removed from interfaces underwater.

In contrast to other underwater adhesion mechanisms, air bubbles adhere better to surfaces with defects, crevices, high roughness, and surface features.^30−33^ This is a result of higher pinning forces at the water–solid–air interface.^34,35^ In addition, a significant area of the bubble performs as a physical gluing surface, which can accommodate the lifting of multiple objects or small particles at once. A major advantage of such a sticky cushion is its flexibility and its performance as a deformable joint.^36^ Namely, the adhesion device is, to some extent, independent of approaching angle and surface topography. On the other hand, adhered objects, such as thin sheets or plates, can be oriented precisely through asymmetric inflation and deflation of arrays of bubbles.

A central aspect of capillary adhesion is the dependence on the surface or interfacial tension.^18,37^ Air capillary bridges generate higher adhesion forces and adhere better to hydrophobic surfaces.^38,39^ Water–air–solid contact lines, however, are highly susceptible to surface defects, which may increase adhesion due to pinning. In addition, for a three-phase contact line to move underwater, water molecules reorganize; therefore, moving contact lines underwater is more energetically consuming in comparison to air.^40^

In this study, we integrate a bubble reversible adhesion mechanism into an automated robotic crane used in submerged environments. We demonstrate the ability to pick and place a variety of small objects underwater and to clean colloidal impurities and particles with arbitrary shapes from submerged surfaces. We address fundamental questions of how to enhance adhesion through contact splitting and what is the role of interfacial energy for underwater adhesion. We demonstrate potential applications of placing and ordering objects in arrays, cleaning submerged surfaces, and reorientating objects underwater.^41^ Finally, we show the possibility to fold thin sheets underwater into three-dimensional structures using the capillary adhesiveness of air bubbles, overcoming the elastic bending energy of the surface.^42,43^

## Results and Discussion

### Robotic Arm Design and Integration of Air Capillary Adhesion Units

**Figure 1:**
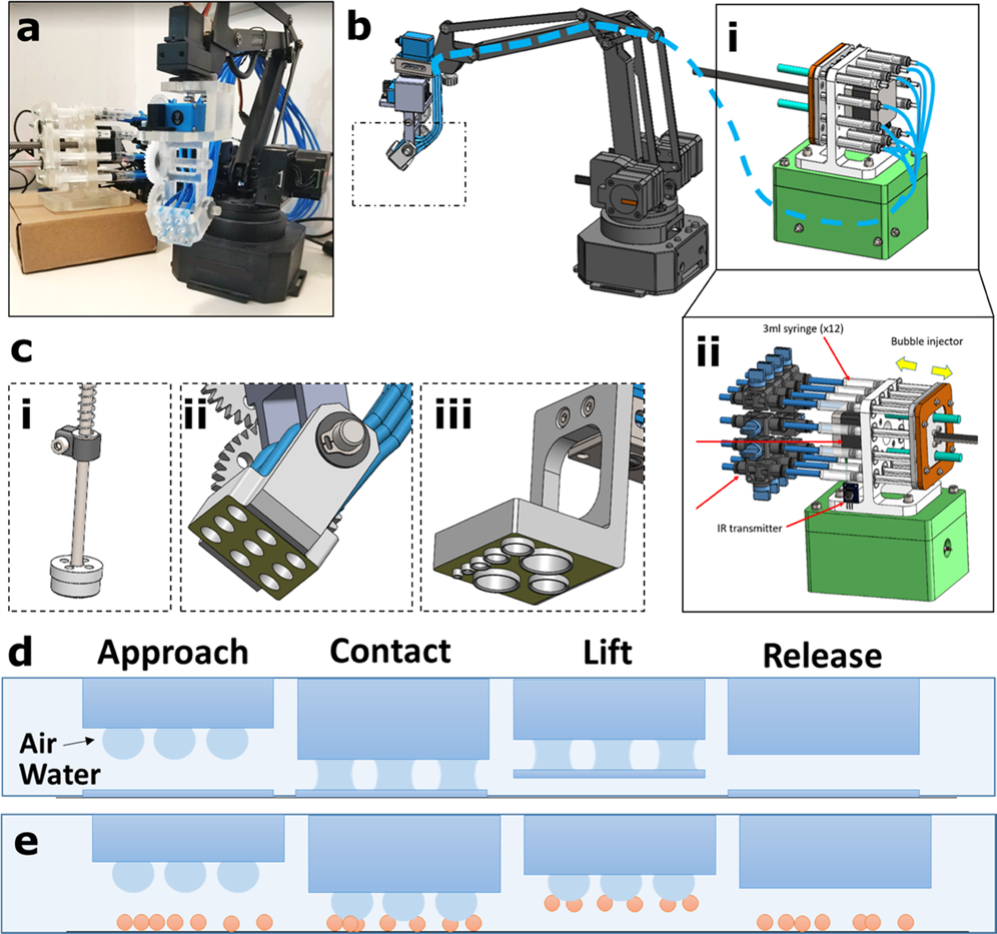
(a) Programmable robotic arm, equipped with an air injection unit to create an array of air capillary bridges. (b) Sketch of the robotic arm. The air supply is marked by a dashed blue line. The motorized injection unit comprises 12 syringes (i), and the airflow is regulated by 12 two-way valves (ii). (c) 3D-printed air injection units: single outlet for pick-and-place tasks (i), an array of nine uniform slits (ii), and injection head with varying sizes of slits (iii). Sketches depicting the adhesion mechanism by creating air capillary bridges between the robotic air injection unit and (d) thin sheets or (e) small particles underwater.

We use a robotic arm with 4 degrees of freedom and repeatability of 0.2 mm (Figure 1a). A syringe-based pump system, including 12 syringes, was constructed to accommodate up to 12 air outlets underwater (Figure 1b). The injection unit is motorized by screw movements to allow reversible injection of air to the air capillary adhesion head (Figure 1b(i)). The number of outlets is modular and controlled by two-way valves to allow the opening and closing of outlets upon request (Figure 1b(ii)). This allows generating multiple air capillary bridges in different arrangements. The injection rate of air is set to 1.5 mL/s, and the maximal volume to 3 mL. Three adhesion heads were designed and 3D printed to fulfill specific tasks (Figure 1c). A single bubble adhesion air-capillary unit was printed to perform pick-and-place tasks (Figure 1c(i)). An adhesion unit of a 3 × 3 array of uniform slits, each 5.1 mm in diameter, was designed to lift thin sheets and particles of varying shapes and to enable alignment of objects using asymmetric inflation of air bubbles (Figure 1c(ii)). In addition, it is used to quantify the adhesion forces, depending on the number of bubbles and their arrangement in an array. Finally, an adhesion unit of varying outlet sizes was printed to establish the scaling law governing the adhesion force, depending on the surface area of the outlet (Figure 1c(iii)).

Sketches showing the physical principle of underwater adhesion derived from air capillary bridges between the robots’ adhesion head and submerged surfaces depict lifting thin sheets (Figure 1d) and small particles (Figure 1e).

Air is injected to create surface-anchored bubbles on the adhesion head. The bubbles are brought close to an object underwater, de-wet, and create adhesive air capillary bridges that are strong enough to lift the object. To release the object, the air is re-sucked into the injection system, the bridges collapse, and the adhesion forces vanish. The typical injection or retraction rates are 1.5 mL/s. These can be further reduced by decreasing the step size of the stepper motor. However, the final state, regardless of the stepper motor velocity, is the complete elimination of the bubble. This results in the release of the held object in all cases.

This principle is used to lift multiple small particles of different geometries and thin sheets. For each case, a different number of bubbles is needed for lifting the object. The contact lines of the air capillary bridges are fixed on the adhesion head and are free to move on the counter side. While the sketch (Figure 1f) depicts spherical particles, the mechanism can be applied to lift a large variety of shapes and geometries of colloids and small objects, as will be described in the following text.

**Figure 2:**
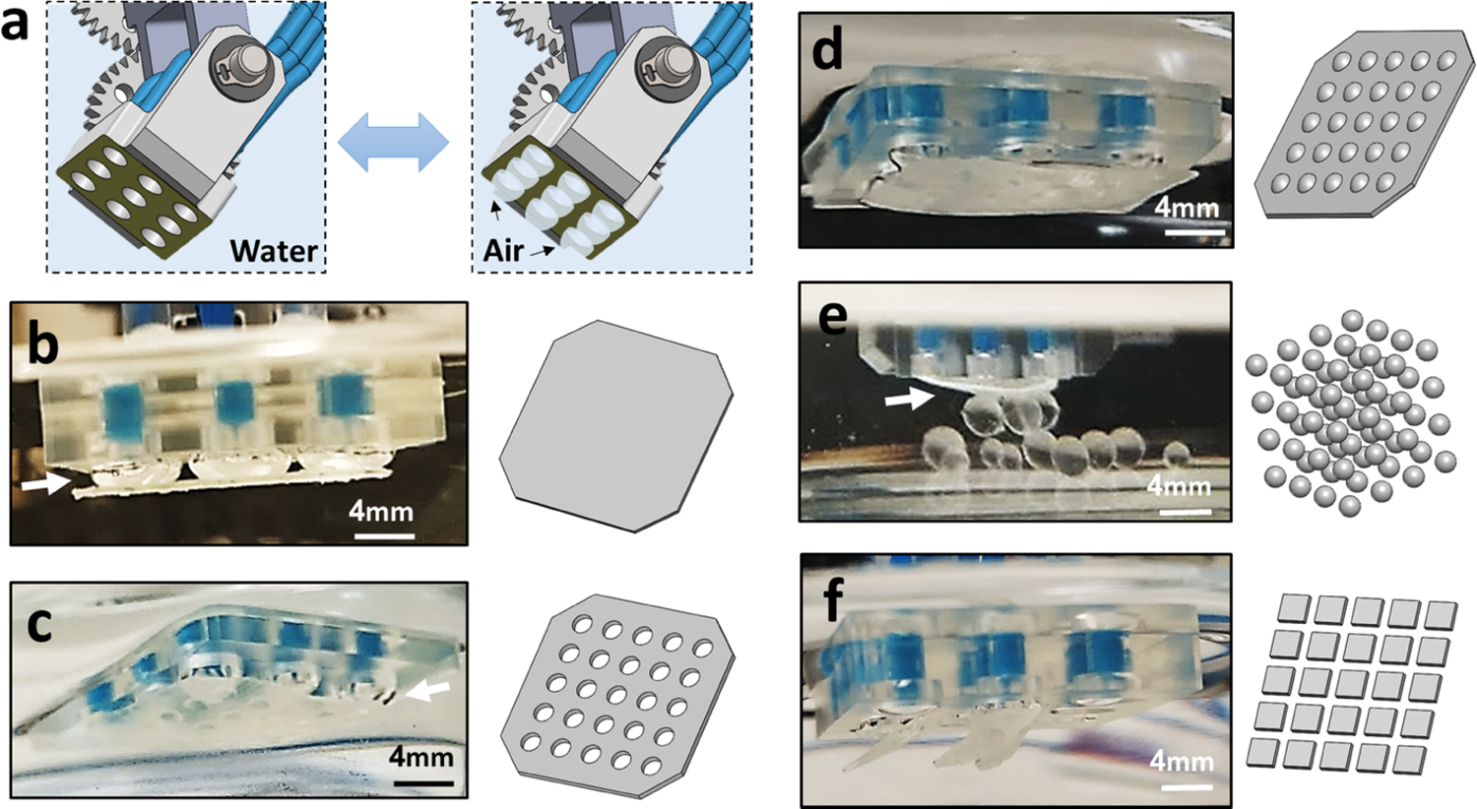
(a) 3 × 3 array of uniform bubbles to create multiple, reversibly inflated, air capillary bridges for lifting (b) a thin continuous 3D-printed polymeric sheet, (c) perforated thin sheet, (d) thin sheet with cavities and bumps, (e) small spheres, and (f) small square particles. The white arrows indicate air capillary bridges, where visible.

A 3 × 3 array of bubbles is formed by the adhesion head shown in Figure 1c(ii). The outlets are independent and create nine uniform reversibly inflated bubbles (Figure 2a). We demonstrate the ability of the bubble array to successfully create capillary bridges with a variety of objects, independent of their shape and morphology.

These objects include a thin, 1 mm thick, 3D-printed continuous polymeric sheet (Figure 2b), perforated thin sheet with holes, 3.5 mm in diameter (Figure 2c), thin sheet with cavities and bumps, 3 mm in diameter (Figure 2d), spheres, 6 mm in diameter (Figure 2e), and square particles with 5 mm edges (Figure 2f). All objects are 3D-printed and have a contact angle of 76 ± 2° (see Sample Preparation section). In cases where the object is larger than the bubble diameter, 5–6 mm each, such as thin sheets, several bridges cooperate to attach and lift the object. On the other hand, when the objects are smaller than the bubble size, a single bubble is sufficient to lift multiple objects simultaneously. In such a case, the number of objects depends on the available air–water interfaces and is regulated by the injected volume.

To assess the adhesion stability of picked objects, we tested the adhesion of a thin sheet (used in Figure 2d) under the rotational motion of the adhesion head at an angular frequency of 0.16 Hz (the maximal frequency enabled by the robotic arm). While some of the contacts with the air capillary bridges were lost, the sheet remained attached even after multiple abrupt motions (see the Supporting Information). The stability of the lifted object depended on the interplay between the adhesion forces derived from the surface hydrophobicity and roughness of the object, and its weight. Within the range of lateral velocities of the robotic arm, ranging between 2 and 5 mm/s, the tested objects in this study were stable against unexpected detachment. While it might be possible to release adhered objects using mechanical vibrations, the most reliable way to release the objects was by eliminating the capillary bridges through suction of the air.

**Figure 3:**
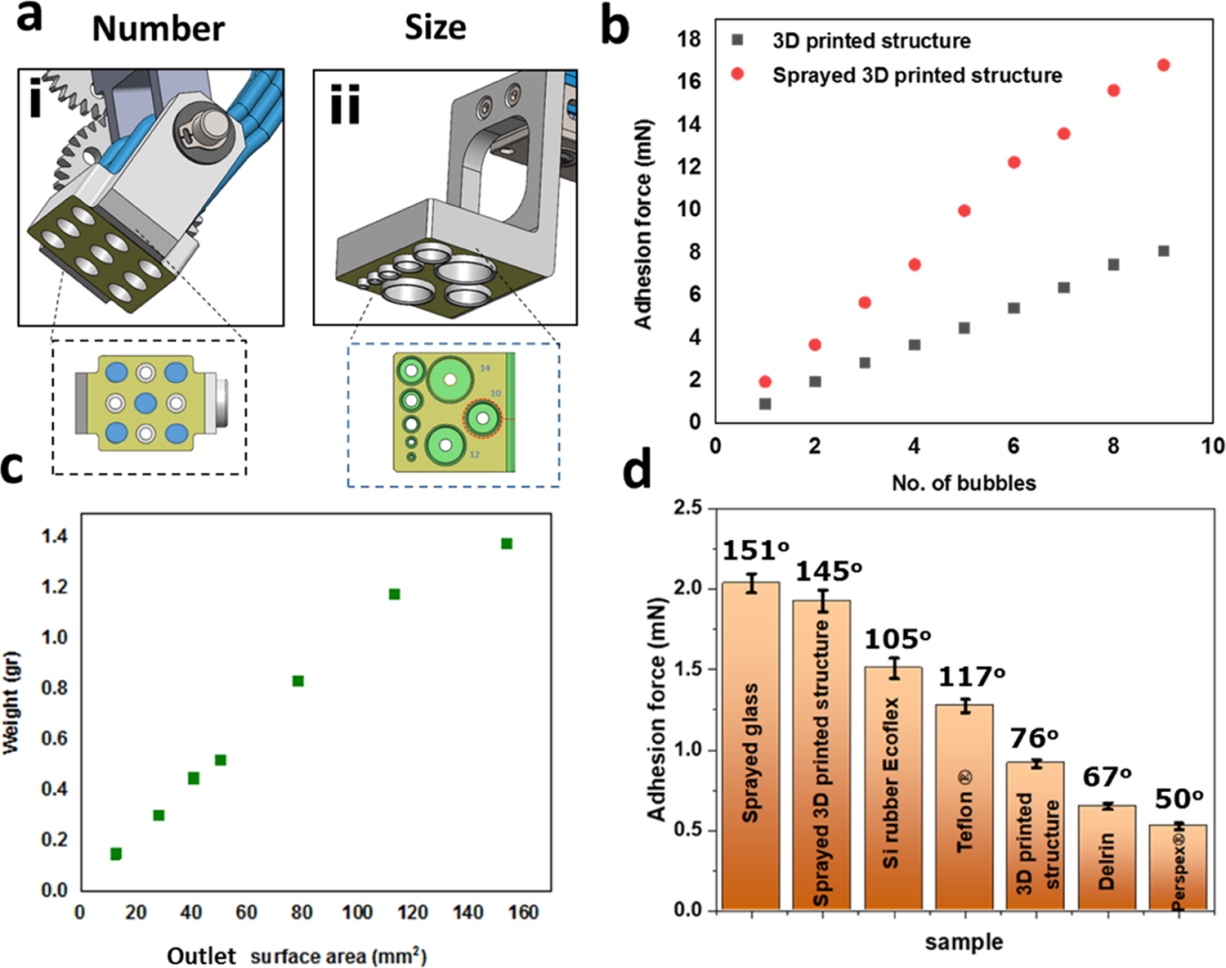
(a) Scheme of the two adhesion heads used to quantify the air-mediated underwater adhesion, depending on (i) number and arrangement of uniform bubbles in a 2D array and (ii) size of the air outlet. (b) Underwater adhesion force, depending on the number of air capillary bridges, used to lift square sheets with CA = 76 ± 2° (black), and the same sheets, coated with a superhydrophobic coating, with CA = 145 ± 2° (red). (c) Lifted weight, depending on the size of the air outlet and consequently the contact area of the air capillary bridge. (d) Underwater adhesion force, depending on the contact angle, for commercially available materials.

To quantify the underwater adhesion strength, we use two adhesion heads. First, the 2D array of uniform bubbles (Figure 3a(i)). Second, an adhesion head with air outlets, varying from 1 to 14 mm in diameter (Figure 3a(ii)). Using the 2D array, several combinations of bubble arrangements are possible. We use nine configurations (Figure S1) to quantify the adhesion force, depending on the number of air capillary bridges. In this experiment, the objects are 3D printed, 30 × 30 mm^2^ squares, ranging from 0.5 to 5.5 g in weight, with CA = 76 ± 2°. We observe a monotonically increasing adhesion force with a linear trend (Figure 3b). We then coat the printed squares with a superhydrophobic coating (see Materials and Methods), resulting in CA = 145 ± 8°. The adhesion force is then doubled, emphasizing the dependence of the mechanism on the hydrophobicity of the objects. The dependence of capillary adhesion on the number of capillary bridges was previously modeled for a large number or liquid capillary bridges, namely, dozens of thousands.^18^ Such large numbers are relevant in nature, where insects use liquid secretion and hairy setae to adhere and adapt to surfaces.^9,44^ However, in smaller numbers, relevant to engineered systems, up to a hundred bridges is a relevant quantity.^22^ We note that adhesion of small insects based on liquid capillary bridges in nature yields forces in the order of a few dozen mN, as measured for the leaf beetle in air and up to a few grams, measured for other beetles (*Hemisphaerota cyanea*), also in the air.^45^ Adhesion of single hairs, creating liquid bridges on the microscale, are in the range of nN.^46^ In an automated device, using a hundred liquid bridges in air, an adhesion force of roughly 39 mN was measured.^22^ In our study, we measure forces of up to 17 mN, using nine bubbles only. These bubbles, however, are roughly 5 times larger in diameter in comparison to the liquid capillary bridges reported previously. We attribute the increased adhesion to several mechanisms. First, pinning forces are much stronger underwater, in comparison with air. This is because water has to be displaced when air–water–solid contact lines advance underwater, in comparison to displacing air when the three-phase contact line advances in air.^47,48^ Second, liquid capillary bridges used to lift objects are subjected to gravitational forces, even if small. On the contrary, bubbles used to create air capillary bridges underwater are subjected to buoyancy forces pointing upward. In such a configuration, the adhesion head inhibits their detachment. This allows the use of larger bubbles in comparison to droplets in air.

Next, we examine the maximal weight that can be lifted by a single air bridge, depending on the outlet surface area (Figure 3c). We choose this parameter since the contact area of the air capillary bridge with the counter surface depends on various factors such as the size, curvature, wetting properties of the object, its surface roughness or defects, and orientation and positioning of the adhesion head. Therefore, we provide guidelines for assessing the adhesion forces depending on the air outlet size. The adhesion head used for this experiment is shown in Figure 3a(ii), with outlet diameters ranging between 1 and 14 mm. Here, as well, we observe a linear trend, with weight ranging between 0.1 and 1.4 g (see Figure S2). It should be noted that when choosing whether to increase the air outlet size versus the number of bubbles, very often the splitting of one bubble into several ones is preferable. Similar to the principle of contact splitting in nature,^49^ which enhances the adjustment to rough surfaces or surface features, the use of several bubbles can not only increase the possible lifted weight but also accommodate objects with a larger variety of shapes. Finally, we quantify the adhesion force, depending on the object contact angle. We use 30 × 30 or 40 × 40 mm^2^ squares with weights ranging from 0.5 to 5 g. Commercially available materials with varying contact angles were tested (see Materials and Methods). As expected, the higher the hydrophobicity, the stronger the adhesion. Note that higher roughness increases pinning and adhesion forces even for hydrophilic materials (CA < 90°). Yet, we observe a monotonically increasing force with increasing the object surface contact angle. The adhesion force to superhydrophobic coated glass (CA = 151 ± 4°) is 3 times larger than that to hydrophilic Perspex with CA = 50 ± 2°. An exception was observed for Teflon with CA = 117 ± 7°, which resulted in lower adhesion in comparison to silicon rubber (Ecoflex) with similar hydrophobicity (CA = 105 ± 3°). We attribute this exception to a relatively smooth surface of the Teflon in comparison to the rubber. In addition, there may be stored elastic energy in rubbery or soft materials that increases the adhesion.^38^ Previous studies, quantifying the traction forces of beetles’ adhesion to solid surfaces underwater via air capillary bridges, yielded forces up to 12 times higher on homogeneous hydrophobic surfaces (CA = 110°) in comparison to smooth hydrophilic surfaces (CA ∼ 40°).^2^ On average, however, these forces were 5 times larger, similar to the enhancement in our system. The air capillary underwater adhesion exhibited by the leaf beetle is derived from six local air plastrons entrapped in the six adhesive pads on the beetle’s feet. Each plastron has an approximate area of 0.04 mm^2^. While these stabilized bubbles are smaller than the mm-scale bubbles used in this study, the enhancement of underwater adhesion depending on the surface hydrophobicity remains similar, implying that similar scaling laws apply in both cases.

**Figure 4:**
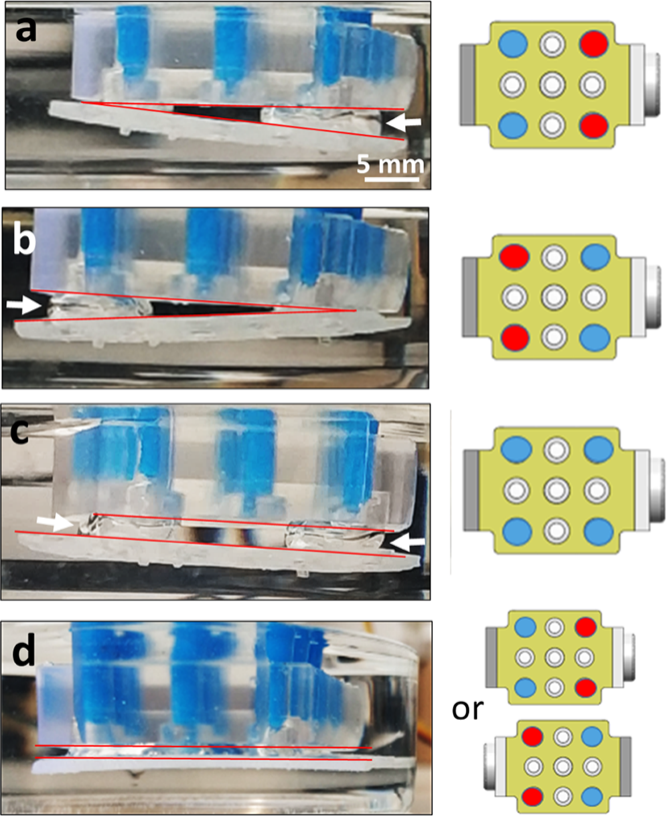
Bubbles acting as mechanical springs. The characteristic length at rest depends on the surface hydrophobicity and volume of the bubble. A thin sheet is aligned underwater, up to an angle of 6°. The alignment is driven by asymmetric inflation and deflation balance (sketches right to the figures) to (a) the right-hand side and (b) left-hand side, respectively. (c) Height of a horizontally leveled sheet is regulated by controlled uniform inflation of the bubbles. The red and blue circles in the sketches indicate inflated and deflated bubbles, respectively. (d) Characteristic length of the air capillary bridges is much shorter for superhydrophobic surfaces, resulting in the loss of the ability to control alignment. The white arrows indicate air capillary bridges, where visible.

Air capillary bridges can be treated as elastic springs, with a characteristic length at rest. The restoring force in this case is a result of the internal Laplace pressure, which can be either negative or positive, resulting in attractive or repulsive forces, respectively. This pressure is dictated by the local radii of curvature in the two major axes of the bridge and the surface tension; in this case, water/air interfacial tension.^50^ Previously, a microrobotic platform, for holding and aligning surfaces on the sub-millimeter scale, is introduced.^51^ Following a similar principle, we induce lateral and angular alignments of lifted objects using controlled inflation and deflation of a two-dimensional array of bubbles (Figure 4).

We examine a thin, 30 × 30 mm^2^, 3D printed sheet with CA = 76 ± 2°. The characteristic length of an air capillary bridge at rest, with a volume of 0.1 mL, corresponds to 2 mm. Figure 4a,b shows the controlled inclination of the thin shit up to 6°, resulting from the asymmetric deflation of air capillary bridges. The red and blue circles in the sketches on the right-hand side indicate inflated and deflated bubbles, respectively. This alignment is fully reversible and can be restored back to a leveled position when the volume of all bubbles in the array is uniform (Figure 4c). Such manipulation is possible for moderately hydrophilic surfaces with CA = 76 ± 2°. The characteristic length of the air capillary bridge becomes smaller with increasing hydrophobicity. Eventually, it is significantly shorter in the case of superhydrophobic surfaces, corresponding to 0.8 mm (Figure 4d), and the ability to asymmetrically inflate and deflate the bubble is lost.

**Figure 5:**
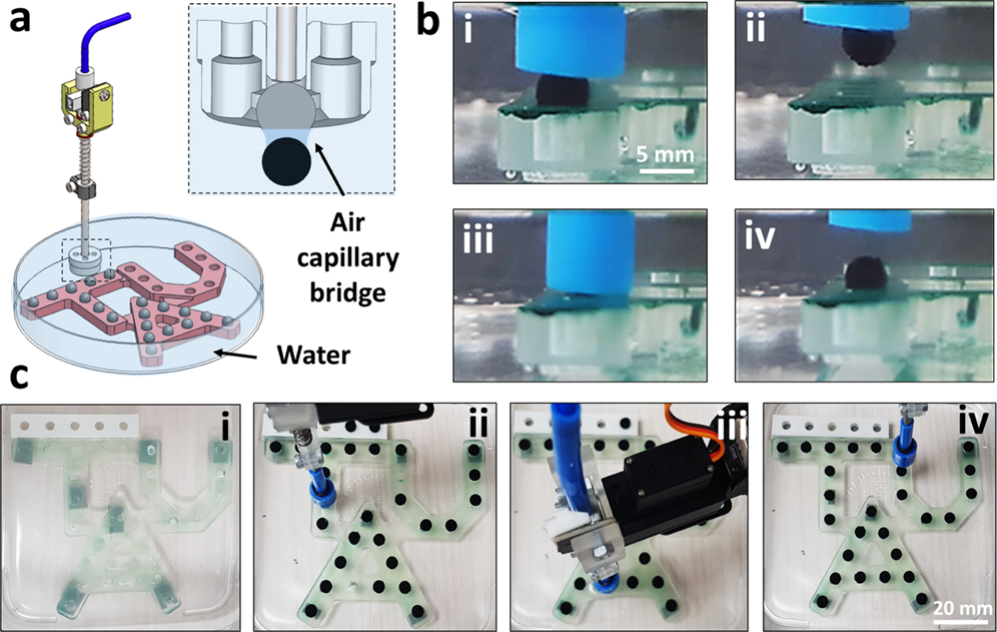
Automated pick-and-place of small polymeric spheres underwater into specific places. (a) Template with 27 cavities is placed underwater. A single bubble adhesive head inflates and deflates an air bubble, creating a deformable air capillary bridge with single spheres. (b) Side view of the pick ((i) and (ii)) and place ((iii) and (iv)) of a sphere through reversible inflation and deflation of the air capillary bridge, respectively. (c) Top view of the template underwater and the automated placement of spheres in precise locations.

We then demonstrate an automated pick-and-place assignment using a single bubble adhesive head (Figure 5). A single air outlet is used to inflate a bubble to a volume of 0.1 mL (Figure 5a). A 3D-printed template with round-shaped cavities is placed at the bottom of a water-filled glass container. For the pick-and-place task, small polymeric spheres (6 mm in diameter) were 3D-printed, with CA = 73 ± 6°, weight ∼0.1 g. They are picked from adjacent storage and are programmed to be transferred into specific locations on the template in a fully automated mode (see Videos 1 and 2). In the inset, the cross section of the adhesive head is seen, together with the air capillary bridge, holding a single sphere. Figure 5b shows the pick-and-place mechanism. The adhesive head with an inflated bubble, 0.1 mL in volume, approaches the sphere (Figure 5b(i)) and creates a contact (Figure 5b(ii)). The head is then programmed to bring the sphere into a specific cavity (Figure 5b(iii)). The air is consequently deflated and the capillary bridge collapses (Figure 5b(iv)). A top view of the entire filling process of the template, with 27 spheres is shown in Figure 5c. The accuracy of the mechanism is dictated by the spatial resolution and repeatability of the robotic arm. In this case, corresponding to 0.2–1 mm.

**Figure 6:**
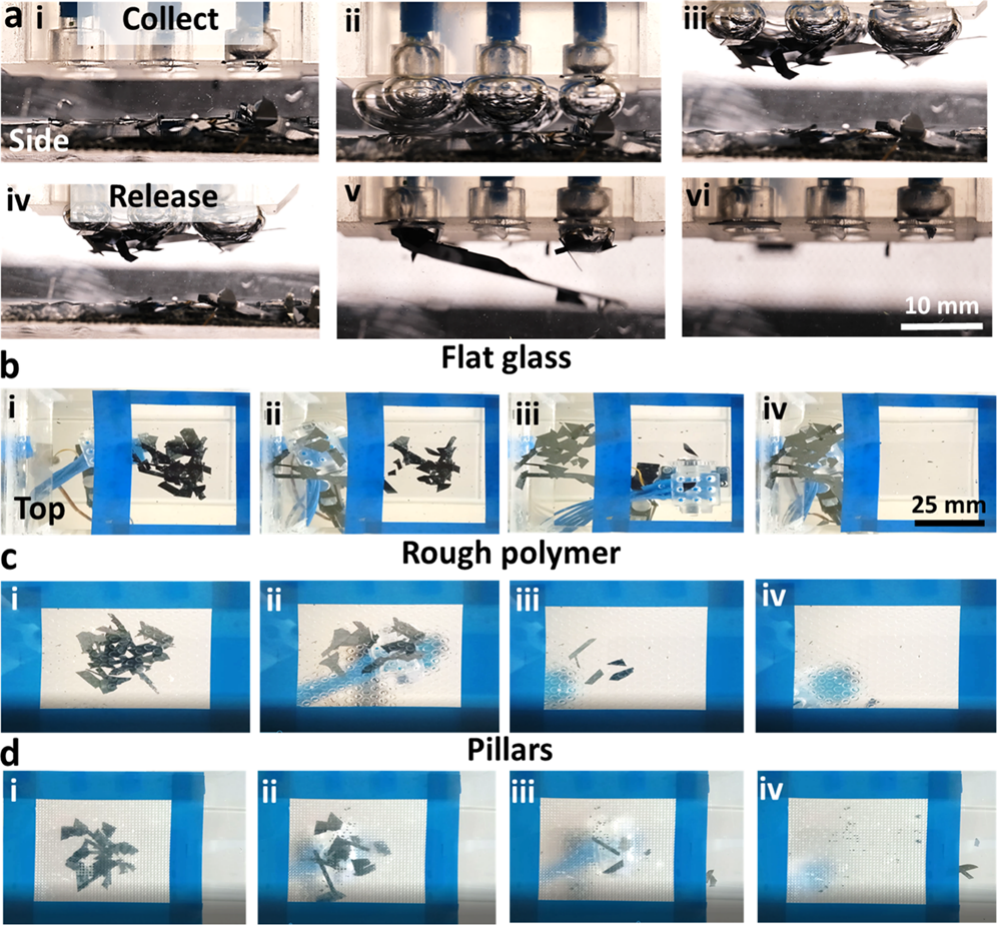
Cleaning surfaces underwater from small hydrophobic contaminants. (a) Side view of air capillary bridges used to lift and release small pieces of Si wafer from a submerged surface. Cleaning silicon pieces from (b) flat glass surface, (c) rough polymeric surface, and (d) polymeric surface covered with a pillar.

Next, we demonstrate the ability to remove contaminants from submerged surfaces via air capillary bridges (Figure 6a). The robotic arm, with a 3 × 3 bubble array head was programmed to move in the cleaning area, marked by blue rectangles (Figure 6b–d).

Pieces of superhydrophobic (CA = 153 ± 5°) Si wafers (see Materials and Methods) were removed from the bottom of either a poly(methyl-methacrylate) (PMMA) container (Figure 6b), a rough polyethylene surface decorated with short elliptical pillars (Figure 6c), or a 3D-printed polymer surface, structured with long round pillars (Figure 6d). See Figure S3 for characterization of the surfaces. Each bubble can accommodate one or multiple particles, depending on the object’s size. In case the object is larger than the bubble diameter, adjacent bubbles may spontaneously cooperate and create multiple air bridges (Figure 6a(iii)). Two main aspects influence the stability of the bubbles in terms of undesired coalescence and detachment from the adhesion head (Video 3): (a) the injection rate and (b) the maximal volume of injected air. Reducing both parameters contributes to the stability of the bubbles against coalescence and detachment; however, this slows down the process and reduces the number of lifted particles per cycle. Therefore, we chose an interplay between these parameters that favor the quick removal of particles.

Note that for cleaning submerged surfaces, an additional mechanism should be further developed to collect the contaminants. One possibility is to lift the adhesion head from the water, resulting in the entrapment of the particles at the water–air interface, from which particles can be further collected. Another possibility is to place particles in a designated container underwater. The fourth degree of freedom in the adhesion head’s motion, allowing tilting, enables us to place particles in such a container. This, however, is beyond the scope of this study.

We note that it is more challenging to remove the Si particles from the flat glass due to high viscous forces between flat surfaces, separated by a thin liquid film, known as Stefan adhesion.^52^ Therefore, rough surfaces or surface features actually facilitate the removal of particles using air capillary bridges, as opposed to other adhesion methods such as suction. In terms of particle size, smaller particles can be also lifted, as demonstrated with brass particles with an average edge length of 264 μm (Figure S4).

**Figure 7:**
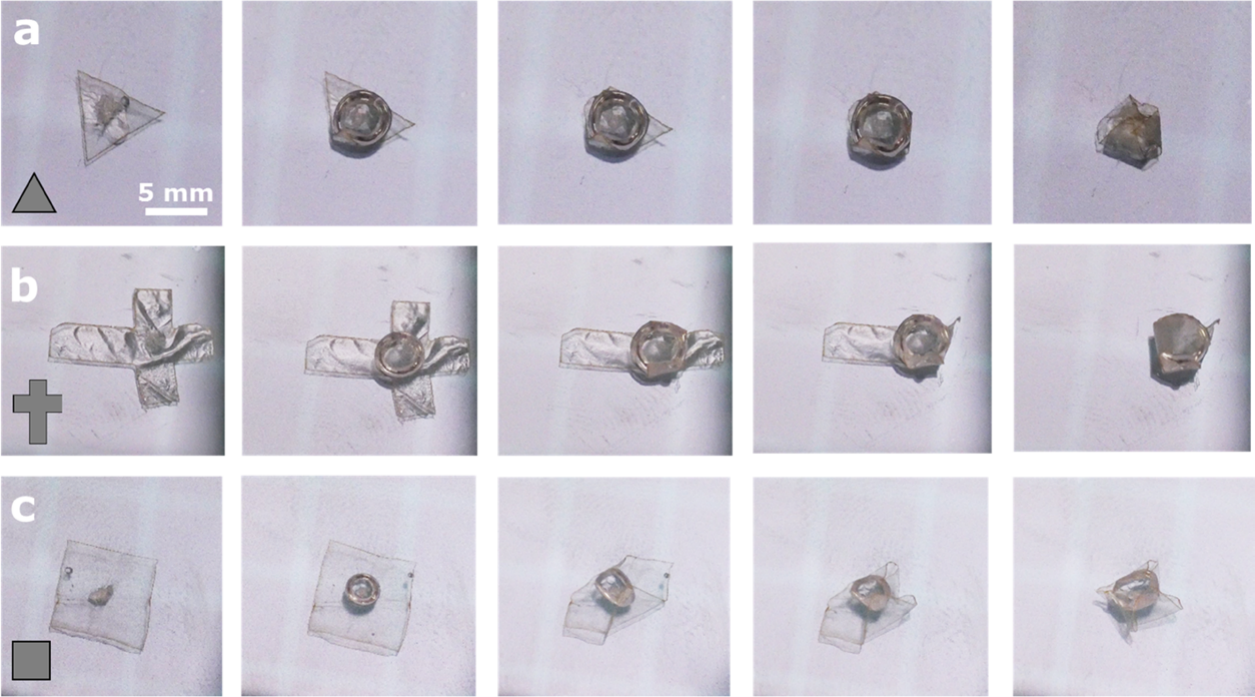
Underwater origami of a thin flexible sheet using air bubbles as a physical glue. (a) Triangular, (b) cross, and (c) square sheets are folded into three-dimensional structures of triangular pyramid, enclosed box, and square pyramid, respectively.

Finally, we demonstrate the ability to create three-dimensional structures underwater by folding thin flexible sheets and fixing their structure using a bubble (Figure 7).

Linear low-density polyethylene (LLDPE) sheets, cut into different geometries, are placed and fixed underwater on the floor of a glass container. A bubble is then placed and moved gently on the surface of the thin sheet. The capillary adhesion forces overcome the elastic bending energy of the sheet and fix it in a three-dimensional folded configuration.^42^ The resulting three-dimensional shape depends on the sheet design: triangular, cross, and square sheets result in triangular pyramid, enclosed box, and square pyramid, as shown in Figure 7a–c, respectively. Alternatively, it is possible to place a bigger bubble to cover the whole area of the sheet and leave the folding process to the shrinkage of the bubble due to diffusion of the gas into the bulk water. This was previously demonstrated for evaporating droplets in the air.^42^ The origami folded structures are stable underwater for up to hours and are limited by the diffusion characteristic time of the gas bubble.^53^

## Conclusions

In summary, we showed that an automated robotic arm, together with ad-hoc air outlet adhesion units, relying on air capillary bridges, can perform as a crane for underwater operations. This includes lifting and aligning of small objects such as thin sheets, spheres, and a variety of particles. We also show the ability to use such configuration for the removal of contaminants from surfaces underwater, to place objects in specific spots on submerged surfaces, and to fold thin sheets into three-dimensional structures. Such adhesion relies on a physical mechanism, as opposed to chemical adhesion, and is fully reversible, does not require adhesive materials, and does not leave traces. We show that although the adhesion is enhanced for hydrophobic and superhydrophobic objects, it can be also used to manipulate moderately hydrophilic surfaces. Such a system can be potentially used to automate cell culture and microfluidics experiments, clean and remove contaminants from submerged surfaces, and manipulate small objects, including placement and alignment underwater.

## Materials and Methods

### Robotic Arm Design and Modification

A four-axis robotic arm (UARM Swift Pro, UFACTORY) was used to conduct the experiments. A motorized air injector powered by a stepper motor, consisting of nine syringes, was designed by CAD software (SolidWorks software) and 3D printed (Formlabs Form 3, MA, using Clear Resin). Twelve shut-off valves were installed to switch off air inputs. Synchronization between the robot axial movements and the air injection unit was achieved by a Python script. An Arduino controller was used to control the injection system and the additional rotated air-injection head.

### Sample Preparation

All objects were designed by SolidWorks CAD software and 3D-printed using Formlabs Form 3 printer and Clear Resin as the ink material unless mentioned otherwise. Samples were washed with isopropyl alcohol (IPA) according to the manufacturer’s protocol and dried in the air.

The commercially available materials or chemically treated surfaces used for the experiments in Figure 3 are described as follows. The fabrication of superhydrophobic samples was done by spray coating (Mud Killer, Joe’s No Flat, Adhestick, Israel) laboratory glass slides. Silicon wafer (Type N, 130 μm, Siltronic, Germany), Perspex (cast, MARGACIPTA WIRASENTOSA, Indonesia), polyoxymethylene (Delrin), silicone rubber (Mold Musk 40, Ecoflex, Smooth-On), Teflon (Scope, Israel), and glass coverslip (Paul Marienfeld GmbH and Co. KG, Lauda-Königshofen, Germany) sprayed by the same superhydrophobic spray.

The spheres in Figure 5 were printed by an extrusion-based method (Flashforge, Creator 3) using polylactic acid filament.

Si wafer (Type N, 130 um, Siltronic, Germany) was crashed into arbitrary pieces (Figure 6) and coated by a superhydrophobic spray (Mud Killer, Joe’s No Flat, Adhestick, Israel) on both sides (CA = 153 ± 5°). The pieces were then placed in a chamber made of PMMA and filled with deionized water. The rough polyethylene surface is decorated with elliptical pillars with 4 mm in the major axis and 3.8 mm in the minor axis. Pillars samples were 3D printed from Clear Resin using a Formlabs 3D printer (see dimensions and surfaces characterization in the Supporting Information, Figure S3).

Linear low-density polyethylene (LLDPE) sheets were cut into different shapes (Figure 7) using a laser cutter (PLS 4.75, Universal Laser Systems). The samples were gently placed and fixed in a Petri dish using a minute amount of glue. The flask was filled with deionized water (DIW).

### Adhesion Force as Function of Bubble Diameter

Disks of different weights (Figure S2) were 3D-printed to evaluate the adhesion force needed to lift samples by varying the air bubble outlet. An air injecting unit was 3D-printed for injecting different sizes of air bubbles.

### Contact Angle Measurements

Contact angles were measured by the static sessile drop method (OCA 20, Data-physics Instruments, GmbH, Filderstadt, Germany). Measurements were performed with distilled water and five drops were used for each sample.

## Supporting Information Available

Representative combinations of bubble arrangements in a 2D array of uniform air outlets (Figure S1), sketch of the 3D-printed weights used to examine the dependence of underwater adhesion on the number of bubbles (Figure S2) and confocal laser scanning microscopy (CLSM) images and illustrations of surface design used for the cleaning experiments (Figure S3), air capillary bridges used to lift small brass particles (Figure S4), and optical images of deionized water contact angle measurements on surfaces used in this study (Figure S5) (PDF)
Pick-and-place tasks from the side view (Video 1) (AVI)
Pick-and-place tasks from the top view (Video 2) (AVI)
Cleaning Si pieces from flat surfaces (Video 3) (AVI)
Cleaning Si pieces from rough surfaces (Video 4) (AVI)
Cleaning Si pieces from pillars covered surfaces (Video 5) (AVI)
Releasing a thin 3D-printed flat sheet through suction of the air and collapse of the air capillary bridges (Video 6) (AVI)
Adhesion of a thin 3D-printed sheet with cavities under a rotational motion of the adhesion head (Video 7) (AVI)

## Supplementary Material

am1c23845_si_001.pdf

am1c23845_si_002.avi

am1c23845_si_003.avi

am1c23845_si_004.avi

am1c23845_si_005.avi

am1c23845_si_006.avi

am1c23845_si_007.avi

am1c23845_si_008.avi
