# Supplementary material for: Automated Manipulation of Miniature Objects Underwater Using Air Capillary Bridges: Pick-and-Place, Surface Cleaning, and Underwater Origami
Source: ACS Appl Mater Interfaces. 2022 Jan 26;14(7):9855–63. doi: 10.1021/acsami.1c23845 (PMC8874901; doi:10.1021/acsami.1c23845)
Supplement: Supplementary file 1 — am1c23845_si_001.pdf [file am1c23845_si_001.pdf]

Supporting Information

Automated Manipulation of Miniature Objects Underwater using Air Capillary Bridges: Pick-and-Place, Surface Cleaning and Underwater Origami

*Tal Weinstein,‡ Hagit Gilon,‡ Or Filc, Camilla Sammartino, Bat-El Pinchasik**

Tel-Aviv University

School of Mechanical Engineering

Faculty of Engineering

6997801

Tel-Aviv, Israel

Corresponding Author

* pinchasik@tauex.tau.ac.il

Video 1. Side view of pick-and-place task of small spheres into a template.

Video 2. Top view of pick-and-place task of small spheres into a template.

Video 3. Cleaning Si pieces from a submerged flat glass surface.

Video 4. Cleaning Si pieces from a submerged 3D-printed rough surface with short pillars (Figure S3 d-f).

Video 5. Cleaning Si pieces from a submerged 3D-printed pillar surface (Figure S3 a-c).

Video 6. Releasing a thin 3D-printed flat sheet through suction of the air and collapse of the air capillary bridges.

Video 7. Adhesion of a thin 3D-printed sheet with cavities under rotational motion of the adhesion head.

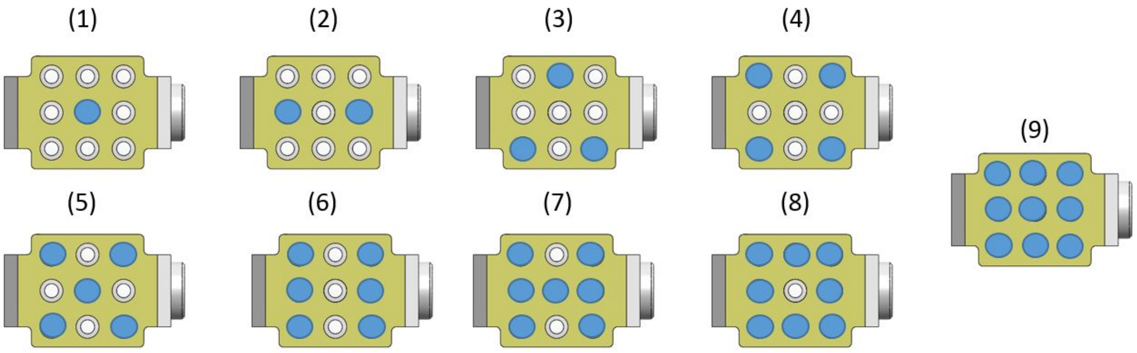

Figure S1. Representative combinations of bubble arrangements in a 2D array of uniform air outlets. The adhesion head corresponds to the one depicted in Figure 1 c(ii).

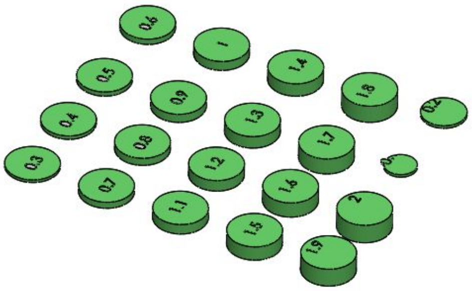

Figure S2. Sketch of the 3D-printed weights, used to examine the dependence of underwater adhesion on the number of bubbles, and consequently air capillary-bridges used to lift them (Figure 3c). All weights have the diameter of 17 mm.

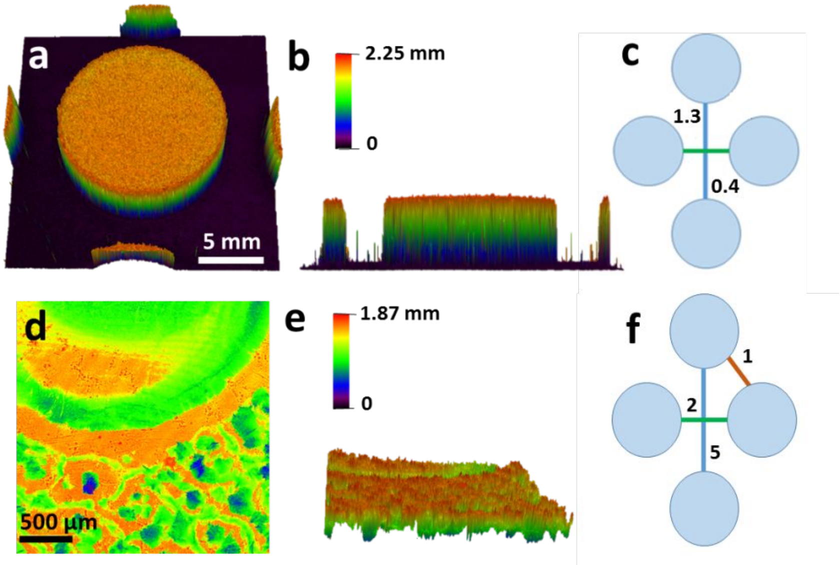

Figure S3. Confocal Laser Scanning Microscopy (CLSM) images and illustrated surface design of the surfaces used in Figure 6 in the manuscript. a) 3D-printed round pillars (top view) and b) side view of the pillars (used in Figure 6d, Video 5 in the supporting information). c) Illustration of the pillars arrangement (top view). Numbers indicate distances in mm. CLSM images of d) top view of the rough surface (used in Figure 6c, Video 4 in the supporting information) and e) side view of the surface. f) Illustration of the pillars arrangement (top view). Numbers indicate distances in mm.

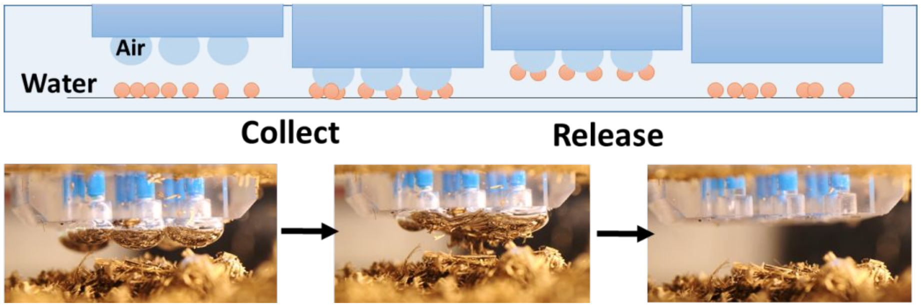

Figure S4. A scheme of air capillary bridges used to lift multiple particles, smaller than the bridge diameter (top) and an experiment with brass particles (bottom). The diameter of the bridges is roughly 5-6 mm, while the average edge length of the particles is 264 µm (and as small as 221 µm). Each bridge accommodates multiple particles.

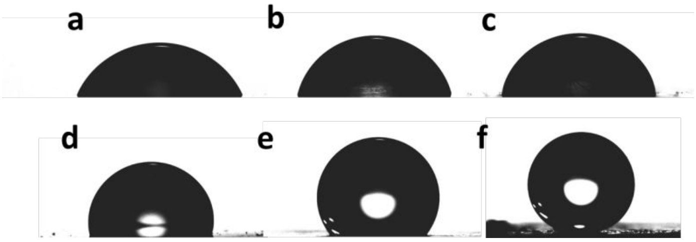

Figure S5. Optical images of deionized water contact angles measurements on surfaces used in this study. a) Perspex (CA= 50 °± 3 °), b) 3D printed Clear Resin® (CA= 76 °± 5 °) c) Delrin (CA= 67 °± 3 °), d) EcoFlex (CA= 105 °± 3 °), e) Teflon (CA= 117 °± 2 °) f) Si wafer, coated with a superhydrophobic coating (CA= 153 °± 5 °). In all measurements, droplets of 2 µl were used.
